# A Proposal for a Structural Model of the Feline Calicivirus Protease Bound to the Substrate Peptide under Physiological Conditions

**DOI:** 10.3389/fmicb.2017.01383

**Published:** 2017-07-25

**Authors:** Masaru Yokoyama, Tomoichiro Oka, Hirotaka Takagi, Hirotatsu Kojima, Takayoshi Okabe, Tetsuo Nagano, Yukinobu Tohya, Hironori Sato

**Affiliations:** ^1^Pathogen Genomics Center, National Institute of Infectious Diseases Tokyo, Japan; ^2^Department of Virology II, National Institute of Infectious Diseases Tokyo, Japan; ^3^Division of Biosafety Control and Research, National Institute of Infectious Diseases Tokyo, Japan; ^4^Drug Discovery Initiative, The University of Tokyo Tokyo, Japan; ^5^Department of Veterinary Medicine, Nihon University Fujisawa, Japan

**Keywords:** feline calicivirus, protease, *in silico* screening, 3-D pharmacophore, BRET technology, MD simulation

## Abstract

Feline calicivirus (FCV) protease functions to cleave viral precursor proteins during productive infection. Previous studies have mapped a protease-coding region and six cleavage sites in viral precursor proteins. However, how the FCV protease interacts with its substrates remains unknown. To gain insights into the interactions, we constructed a molecular model of the FCV protease bound with the octapeptide containing a cleavage site of the capsid precursor protein by homology modeling and docking simulation. The complex model was used to screen for the substrate mimic from a chemical library by pharmacophore-based *in silico* screening. With this structure-based approach, we identified a compound that has physicochemical features and arrangement of the P3 and P4 sites of the substrate in the protease, is predicted to bind to FCV proteases in a mode similar to that of the authentic substrate, and has the ability to inhibit viral protease activity *in vitro* and in the cells, and to suppress viral replication in FCV-infected cells. The complex model was further subjected to molecular dynamics simulation to refine the enzyme-substrate interactions in solution. The simulation along with a variation study predicted that the authentic substrate and anti-FCV compound share a highly conserved binding site. These results suggest the validity of our *in silico* model for elucidating protease-substrate interactions during FCV replication and for developing antivirals.

## Introduction

Feline calicivirus (FCV) is a positive-strand, non-enveloped RNA virus belonging to the genus *Vesivirus* in the family *Caliciviridae* (Green et al., 2000). The virus is a major cause of the upper respiratory tract disease in cats. In addition to its importance in veterinary health, FCV is invaluable to studies of the molecular biology of calicivirus, because it is cultivatable in feline cells (Pesavento et al., 2008), and the reverse genetics systems are available (Sosnovtsev and Green, 1995; Oka et al., 2014; Sandoval-Jaime et al., 2015). The FCV genome is an approximately 7.7 kb positive-sense, single-stranded RNA with three open reading frames (ORFs). ORF1 encodes non-structural proteins, while ORF2 and ORF3 encode structural proteins, including the precursor capsid and minor structural proteins, respectively (Pesavento et al., 2008). FCV protease is initially synthesized as a part of the ORF1 polyprotein. The protease cleaves ORF1 and ORF2 precursor proteins to achieve productive infection in infected cells (Sosnovtsev et al., 1998, 2002). Thus, the study of interactions between FCV protease and its substrates is critical for understanding the molecular steps of viral replication and for developing antivirals.

The viral protease activity of FCV resides in the carboxy-terminal region of the ORF1-encoded precursor protein (Sosnovtseva et al., 1999; Oka et al., 2007), whereas six sites for the protease-dependent cleavage are distributed across the ORF1 and ORF2-encoded proteins (Sosnovtsev et al., 2002). A glutamic acid residue, which is located immediately upstream of the peptide bond subjected to the hydrolysis, is named the P1 site and is highly conserved among the six cleavage sites and among the distinct FCV strains (Oka et al., 2009), suggesting its essential role in the cleavage reaction. Consistently, an E to A substitution of the P1 site has been shown to result in the elimination of precursor susceptibility to viral protease (Sosnovtsev et al., 2002; Oka et al., 2009). Interestingly, however, the amino acid residues located upstream of the P1 site are variable among sites and among FCV strains, and amino acid substitutions are tolerated by the FCV protease although processing efficiency could be modulated by mutation at the P3 and P4 sites (Oka et al., 2009). An essential step toward elucidating the roles of the variation in FCV replication and evolution is thus to achieve a structural understanding of the interactions between viral protease and its substrates. However, how FCV protease interacts with its substrates remains unknown due to a lack of structural information on the protease-substrate complex.

To address this issue, we constructed a molecular model of the FCV protease bound with the octapeptide, which corresponded to a cleavage site of the FCV VP1 capsid precursor protein, by using homology modeling and docking simulation. The model was used to characterize the structure of the substrate in the protease-bound state and to screen for a molecule that mimics the structural properties and arrangement of substrate. The *in silico* search, in combination with bioluminescence technique for assessing the FCV protease activity in the FCV-infected cells (Oka et al., 2011), led to identification of a small molecule that structurally resembles parts of the substrate in the protease-bound state and has anti-FCV activities in the infected cells. The MD simulation along with a variation study using Shannon entropy (Motomura et al., 2008; Naganawa et al., 2008; Oka et al., 2009) indicated that the authentic substrate and the substrate-mimic share the highly conserved binding site. The consistency of the *in silico* screening and experimental results suggests the validity of our *in silico* model for elucidating protease-substrate interactions during viral replication.

## Materials and Methods

### Viruses and Cells

Two FCV strains were used for the present study. The FCV F4 strain was isolated in Japan from a cat with signs of respiratory tract infection (Takahashi et al., 1971), whereas the FCV 2280 strain was isolated in the United States from the American Tissue Culture Collection (Oka et al., 2013). Crandell Rees feline kidney (CRFK) cells were obtained from the Japanese Collection of Research Bioresources and cultured in Eagle’s minimal essential medium (MEM; Sigma–Aldrich, St. Louis, MO) supplemented with 5% heat-inactivated fetal bovine serum (Invitrogen, Carlsbad, CA, United States) and antibiotics (100 U/mL penicillin and 100 g/mL streptomycin; Invitrogen). The viruses were propagated and titrated in CRFK cells with a conventional assay as described previously (Oka et al., 2011).

### Chemical Compound Library

Chemical compounds were obtained from the Open Innovation Center for Drug Discovery (The University of Tokyo, Tokyo, Japan) as described previously (Yokoyama et al., 2012b). The database of this center provides information on the molecular formula and structure, molecular weight, hydrogen-bond donor-acceptor numbers, topological polar surface area, and other physicochemical parameters of the compounds for pharmacophore-based *in silico* drug screening.

### Molecular Modeling of a FCV Protease Docked to a Substrate Octapeptide

A molecular model of a ligand-free protease domain (154 aa) of the FCV F4 strain isolated in Japan (Oshikamo et al., 1994; Oka et al., 2007) (GenBank accession number: D31836) was constructed by homology modeling using the tools available in the Molecular Operating Environment (MOE; Chemical Computing Group Inc., Montreal, QC, Canada). As the modeling template for the FCV protease domain, we used the high-resolution crystal structure of Norwalk virus 3C-like protease at a resolution of 1.50Å (PDB code: 2FYQ) (Zeitler et al., 2006). The thermodynamically and physically optimized protease model was used to construct the protease-substrate complex model. The octapeptide (F^P4^R^P3^L^P2^E^P1^/A^P1^′D^P2^′D^P3^′G^P4^′) corresponding to the authentic cleavage site of the FCV F4 capsid precursor was constructed by using the Molecular Builder tool in MOE. The octapeptide was docked with the optimized FCV protease domain model described above, using the automated ligand docking program ASEDock2005 (Goto et al., 2008) operated in MOE. The default settings in ASEDock2005 were applied to search for the candidate docking structures, and the structures with the best docking score expressed by the arbitrary docking energy (U_dock_) in ASEDock2005 were selected for the analysis of the protease-substrate interaction sites.

### Pharmacophore-Based *In Silico* Screening

Chemical compounds that have some structural similarity with the cleavage site of the FCV F4 strain ORF2 (P1-P4 sites: amino acid numbers 121–124 (Oka et al., 2009) were searched by pharmacophore-based *in silico* screening using tools available in the MOE. We created a pharmacophore query with a substrate feature using the Pharmacophore Query Editor tool in MOE. Pharmacophore-based *in silico* screening was done by the Pharmacophore Search module in the MOE using the created query as described previously (Yokoyama et al., 2012b).

### Cell-Free FCV Protease Activity Assay

The susceptibility of FCV protease to the hit compound was analyzed by an *in vitro trans* cleavage assay essentially as described previously (Oka et al., 2009). We tested compound No. 108 and its derivative having the portion of the compound No. 108 (compound No. 731). A radiolabeled C-terminus six histidine (His) tagged FCV F4 capsid precursor, or a non-radiolabeled N-terminus six His-tagged FCV F4 NS6-7 containing protease and RNA dependent RNA polymerase (RdRp) region (Sosnovtseva et al., 1999; Oka et al., 2007) was separately expressed using the TNT T7 Quick for PCR DNA kit (Promega, Madison, WI, United States). The primers used to generate linear DNA fragments for C-terminus six His-tagged FCV F4 capsid precursor protein are 5′-GGATCC
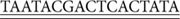
GGGAACAGCCACCATGTGCTCAACCTGCGCTAACGTG-3′ including T7 promoter (double underlined) and 5′-T_30_TTAgtgatggtgatggtgatgTAATTTAGTCATTCTGCTCCTAATG-3′ including His-tag sequence (lower cases). The primers used to generate linear DNA fragments for N-terminus six His-tagged FCV F4 NS6-7 fusion protein are 5′-GGATCC
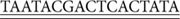
GGGAACAGCCACCATGcatcaccatcaccatcacTCTGGGCCTGGCACTAAATTTC-3′ including T7 promoter (double underlined) and His-tag sequence (lower cases), and 5′- T_30_TTAAACTTCGAACACATCACAGTG-3′. PCR was performed with 100 ng of pUC19/FCV F4 plasmid DNA that contains the full-length FCV F4 cDNA as previously described (Oka et al., 2007). To express radiolabeled polyproteins, 3 μl of the PCR mixture was mixed with 20 μl of TNT T7 PCR Quick Master Mix (Promega) and 2 μl of EasyTag [^35^S] methionine (PerkinElmer Japan, Kanagawa, Japan), and the mixture was incubated at 30°C for 3 h. To express the non-radiolabeled polyproteins, 5 μl of the PCR mixture was mixed with 40 μl of TNT T7 PCR Quick Master Mix (Promega), 1 μl of 1 mM Methionine (Promega), and 4 μl of nuclease free water, and the resultant mixture was incubated at 30°C for 3 h. The His-tagged FCV proteins (capsid precursor and NS6-7) were purified from reaction mixture using MagZ Protein Purification System (Promega) according to the manufacture’s instruction. To examine the cleavage activity of FCV protease in *trans*, 20 μl of the freshly prepared non-radiolabeled FCV NS6-7 was mixed with 0–4 μl of compound No. 108, or No. 731 in DMSO (final concentration of 0, 164, 323, 625, and 1,176 μM) and 10 μl of the freshly prepared radiolabeled capsid precursor protein. The mixtures were incubated at 30°C for 20 h and separated by SDS-polyacrylamide gel electrophoresis (SDS-PAGE). In brief, 4 μl of the reaction mixture was separated by SDS-PAGE and the proteins in the gel were blotted onto a polyvinylidene difluoride membrane (Immobilon-P; Millipore, Tokyo, Japan) with a semi-dry electroblotting apparatus (ATTO, Tokyo, Japan). Radiolabeled proteins were detected with a Typhoon FLA 7000 (GE HealthCare UK, Little Chalfont, United Kingdom).

### Cell-Based FCV Protease Assay

The FCV protease inhibitory activity of the 133 candidate compounds was evaluated by means of a cell-based biosensor assay using the principle of second-generation bioluminescence resonance energy transfer (BRET^2^) principle (Oka et al., 2011). CRFK cells constitutively express a biosensor recombinant protein, BRET^2^-FCV-Cut, harboring an FCV protease recognition sequence, PLFRLE/ADDGSI, corresponding to the P6–P6′ positions of the cleavage site in the precursor capsid protein of the FCV F4 strain inserted between the donor (Renilla luciferase) and acceptor (GFP^2^) of a BRET^2^ pair (Oka et al., 2011). The BRET^2^-FCV-Cut-stably expressing CRFK cells were cultured in 96-well plates (ViewPlate white-96; PerkinElmer). The culture medium was then aspirated, and 100 μL of medium without serum containing FCV F4 strain was added with an approximate multiplicity of infection (MOI) of 10. The same medium without serum and FCV was used for the negative control. After 1 h of infection, 10 μL of phosphate buffered saline (PBS) containing 500 or 400 μM chemical compound was added (final concentration of 45.4 μM or 36.4 μM). At ∼6 h post-infection with FCV, the medium was aspirated, and washed once with 250 μL of MEM-alpha (Invitrogen). The BRET^2^ signal was detected by sequential dual luminescence measurements mode for each well immediately after adding 50 μL of MEM-alpha containing 2 ng/μL Coelenterazine 400A (Biotium Inc., Hayward, CA, United States) by using a 2030 Multilabel Reader ARVO-X3 system linked to a liquid dispenser. The BRET^2^ signal ratio was calculated as the GFP^2^ emission (515 nm) divided by the RLuc emission (410 nm). The CRFK cells expressing BRET^2^-FCV-Cut showed a BRET^2^ ratio of approximately 1.0 without FCV infection, whereas CRFK cells expressing BRET^2^-FCV-Cut exhibited declining BRET^2^ ratios (GFP^2^/ RLuc) when the FCV protease cleaved the recognition sequence, because the GFP^2^ and RLuc proteins drifted away from each other, and the energy transfer from RLuc to GFP^2^ was lost. In this study, compounds that showed the same level of BRET^2^ signals as the FCV non-infected well (approximately 1.0 or above) were selected as hit compounds for further analysis.

### Assessment of Antiviral and Cytotoxic Effects of Compound No. 108

Confluent monolayers of CRFK cells in 6-well plates (approximately 10^6^ cells/well) were inoculated at a MOI of 0.0001 with the FCV F4 strain or FCV 2280 strain isolated in the United States (Oka et al., 2013) (Genbank accession no. KC835209) and then incubated for 2 h. The cells were washed once with PBS without Ca^2+^and Mg^2+^ [PBS (-)] and incubated in 2 mL of serum free medium including compound No. 108 (25 or 12.5 μM) or DMSO at 37°C up to 40 h. The virus titers in the supernatant were determined by the 50% tissue culture infective dose (TCID_50_) method as described previously (Oka et al., 2011). For evaluation of the cytotoxic effect, the CRFK cells were cultured in the presence of increasing concentrations of a given chemical compound for 48 h. The cell variability was quantified by a CellTiter Glo Luminescent Cell Viability Assay Kit (Promega) according to the manufacturer’s instructions. ATP-dependent luminescence signals were measured with a 2030 Multilabel Reader ARVO-X3.

### Structural Modeling of FCV Protease Docked to Compound No. 108

Candidate chemical compounds were docked with the FCV F4 or FCV 2280 strain protease domain model using the automated ligand docking program ASEDock 2005 (Goto et al., 2008) operated in MOE as described previously (Yokoyama et al., 2012b). The default settings in ASEDock2005 were applied to search for the candidate docking structures, and the structures with the best docking score expressed by the arbitrary docking energy (U_dock_) in ASEDock2005 were selected for the analysis of protease-ligand interaction sites.

### MD Simulation of FCV Protease Docked to the Substrate Octapeptide

The molecular model of the FCV F4 strain protease-substrate complex was subjected to MD simulation essentially as described for simulations of the HIV-1/SIV envelope gp120 subunit (Naganawa et al., 2008; Yokoyama et al., 2012a, 2016; Kuwata et al., 2013; Hikichi et al., 2016). Briefly, the MD simulation was performed by the Particle Mesh Ewald Molecular Dynamics (PMEMD) module in the AMBER program package (Case et al., 2014), employing the Amber ff99SB-ILDN force field, a protein force field with improved side-chain torsion potentials (Lindorff-Larsen et al., 2010), and the TIP3P water model for simulation of aqueous solutions (Jorgensen et al., 1983). Bond lengths involving hydrogen were constrained with SHAKE, a constraint algorithm to satisfy a Newtonian motion (Ryckaert et al., 1977), and the time step for MD simulation was set to 2 fs. A non-bonded cutoff of 10 Å was used. After heating calculations for 20 ps until 310 K using the NVT ensemble for the constant volume, temperature, and numbers of particles in the system, the simulation was executed using the NPT ensemble for the constant pressure, temperature, and numbers of particles in the system at 1 atm, at 310 K, and in 150 mM NaCl for 30 ns. The protease was restrained using a harmonic potential with a graded decreasing force constant from 10 to 1 kcal/mol/Å^2^.

### Analysis of Individual Amino Acid Variation

The amino acid sequences of the protease domain (154 amino acid residues in length) (Sosnovtseva et al., 1999; Oka et al., 2007) of 15 FCV strains for which complete genome sequences were available as of 2013 June (Oka et al., 2013) were obtained from GenBank (accession numbers: D31836, AF479590, U13992, M86379, AF109465, L40021, AY560118, AY560116, AY560117, AY560113, AY560114, AY560115, DQ424892, GU214989, and KC835209). Amino acid variations at each position of the FCV protease domain [amino acid numbers 1072–1225 (Oka et al., 2007)] were calculated with a multiple sequence alignment as described previously for other viral proteins (Motomura et al., 2008; Naganawa et al., 2008; Oka et al., 2009) on the basis of Shannon’s equation (Shannon, 1997):

$$\begin{array}{c} H\left(i\right)=-\sum_{x_{i}}p\left(x_{i}\right)\log p\left(x_{i}\right)(x_{i}=G,A,I,V,\;......\;), \end{array}$$

where *H(i), p(x*_i_*)*, and *i* indicate the amino acid entropy score of a given position, the probability of occurrence of a given amino acid at the position, and the number of positions, respectively. An *H(i)* score of zero indicates absolute conservation, whereas 4.4 bits for amino acids indicate complete randomness. For analysis of the diversity in the chemical properties, the amino acid residues were classified into seven groups: acidic (D, E), basic (R, K, H), neutral hydrophilic (S, T, N, Q), aliphatic (G, A, V, I, L, M), aromatic (F, Y, W), thiol-containing (C), and imine (P). For analysis of the diversity in the size of side chains, the amino acid residues were classified into 4 groups: small (G, A, C, S), medium-small (T, V, N, D, I, L, P, M), medium-large (Q, E, R, K), and large (H, F, Y, W) (Oka et al., 2009; Yokoyama et al., 2012b).

## Results

### Pharmacophore-Based *In Silico* Screening for the Substrate Mimic

**FIGURE 1:**
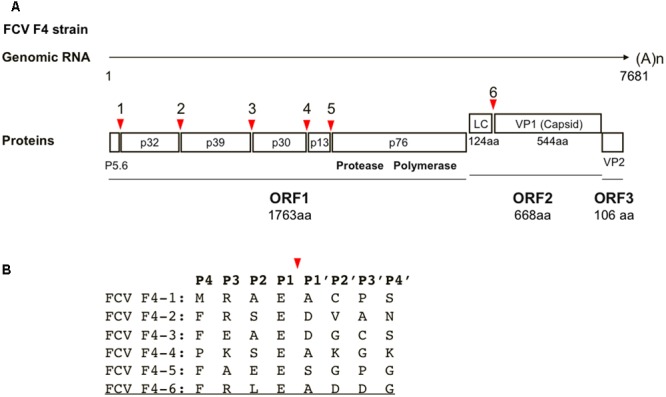
Cleavage map of FCV precursor proteins and sequences around cleavage sites. **(A)** Cleavage map of the precursor proteins of the FCV. Red arrows illustrate the locations of five cleavage sites in ORF1 and a single cleavage site in ORF2 (Sosnovtsev et al., 2002). The map is based on the RNA genome information of the FCV F4 strain (Oshikamo et al., 1994). **(B)** Amino acid sequences around cleavage sites. The P4–P4′ sequences of the six cleavage sites are indicated by a single letter code. A red arrow shows the cleavage site. The octapeptide used in this study is underlined.

The three-dimensional (3-D) structure of the FCV protease-substrate complex is an important tool for investigating protease-substrate interactions for viral replication and for developing antivirals. However, this information is not yet available. Therefore, we constructed a molecular model of the FCV protease docked to its substrate. We chose the protease of the FCV F4 strain (Oshikamo et al., 1994) for our present structure-based inhibitor search, largely because *in silico* and experimental information and reagents are available for this protease, including the structural features of the catalytic sites of protease (Oka et al., 2007), functional information on the processing sites (Oka et al., 2009), the protease assay system using the cells (Oka et al., 2011), and the infectious molecular clone (Oka et al., 2014). **Figure 1A** shows the cleavage map of the precursor proteins of the FCV F4 strain. Among the six cleavage sites, only the P1 site of is highly conserved in the F4 strain, whereas variation exists at the other P4–P4′ cleavage sites (**Figure 1B**). An octapeptide (F^P4^R^P3^L^P2^E^P1^/A^P1^′D^P2^′D^P3^′G^P4^′) corresponding to the cleavage site for generating the mature capsid protein was used as a substrate mimic for screening the FCV inhibitor, because the presence of a long leader sequence (about 125 amino acids in length) of the capsid precursor protein is unique to genus *Vesivirus* in the family *Caliciviridae* (Green et al., 2000) and essential for FCV replication (Sosnovtsev et al., 1998). A molecular model of the protease docked to the FCV FV4 octapeptide was constructed by homology modeling and docking simulation using the tools available in MOE (see Materials and Methods for details).

**FIGURE 2:**
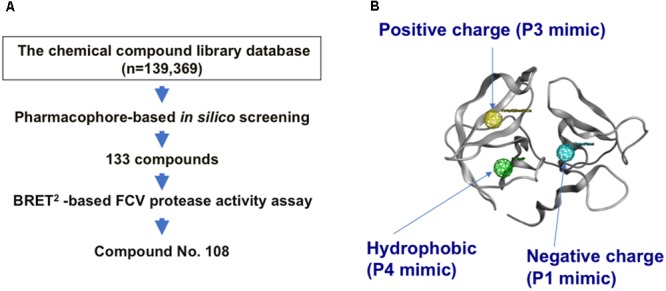
Pharmacophore-based *in silico* screening for the substrate mimic. **(A)** Flow chart for *in silico* screening. A structural model of the FCV protease bound to the FCV F4-6 peptide was constructed by homology modeling and docking simulation. Using the complex model, pharmacophore-based *in silico* screening (Schuster et al., 2006a,b; Kirchmair et al., 2007) was applied in combination with a cell-based protease activity assay to identify a compound, named No. 108, from a chemical library containing 139,369 compounds. The No. 108 has structural features of an authentic substrate in the protease-bound state and has the ability to inhibit FCV protease activity and viral infectivity in the infected cells **(Figures 3, 4)**. **(B)** Pharmacophore mapping on the FCV protease model. An aromatic-ring-like portion resembling the P4 amino acid residue, a positively charged portion resembling the P3 amino acid residue, and a negatively charged portion resembling the P1 amino acid residue are arranged at 3-D positions similar to those in the authentic substrate in the FCV protease.

Using the complex model, we conducted a pharmacophore-based *in silico* screening of the FCV substrate mimics (**Figure 2A**), as was done in the search for the anti-sapovirus compounds (Yokoyama et al., 2012b). Pharmacophore-based *in silico* screening is a computational screening method for the candidate molecules that fit structures and arrangements of molecular portions required for ligand interactions, i.e., pharmacophore (Schuster et al., 2006a,b; Kirchmair et al., 2007). A total of 139,369 chemical compounds (molecular weights 42–2986) were screened for the molecules containing an aromatic-ring-like portion resembling the P4 amino acid residue, a positively charged portion resembling the P3 amino acid residue, and a negatively charged portion resembling the P1 amino acid residue, with each being arranged in a 3-D position similar to that of the authentic substrate (**Figure 2B**). Using the protease-ligand structure-based *in silico* screening, we identified a total of 133 compounds that fit the pharmacophore features of the authentic substrate octapeptide bound into the protease.

### Evaluation of Anti-FCV Activity of the Candidate Molecules

**FIGURE 3:**
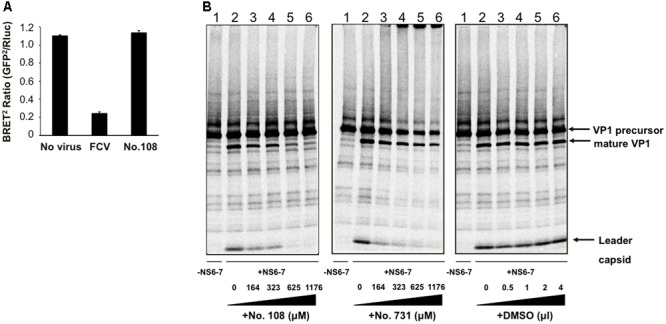
Evaluation of anti-FCV protease activity of the candidate molecules. **(A)** BRET^2^ -based FCV protease activity assay (Oka et al., 2011). BRET^2^-FCV-Cut- expressing CRFK cells (Oka et al., 2011) were infected with the FCV F4 strain at an MOI of 10, and incubated for 6 h. BRET^2^ signals in FCV non-infected cells, FCV-infected cells, and FCV-infected cells in the presence of the compound No. 108 (45 μM) were measured. The BRET^2^ ratio was defined as the intensity of GFP^2^ emission at 515 nm divided by that of Rluc at 410 nm. Error bars represent the standard error of the mean (*n* = 2). **(B)** *In vitro* proteolysis assay. [^35^S]-labeled, His-tagged VP1 precursor protein of the FCV F4 strain and non-radiolabeled His-tagged NS6-7 protein containing protease of F4 strain (Oka et al., 2007) were separately expressed using *in vitro* transcription-translation system (Oka et al., 2009). Translational products were then purified with MagZ Protein Purification System (Promega) and mixed. To examine the effects of candidate compounds, 0–4 μl of No. 108 (left panel), No. 731 (middle panel), or DMSO (right panel) was added into 30 μl of the substrate-protease mixture, and the mixtures were incubated at 30°C for 20 h and separated by SDS-polyacrylamide gel electrophoresis (Oka et al., 2009).

A BRET^2^-based FCV protease activity assay (Oka et al., 2011) was used to identify the anti-FCV compound No. 108 from the 133 molecules selected *in silico*. The BRET^2^ ratio was approximately 1.1 without FCV protease activity (**Figure 3A**, No virus), whereas FCV infection caused an approximately five-fold reduction in the BRET^2^ ratio due to the cleavage of an FCV specific substrate for the second-generation bioluminescence resonance energy transfer (Oka et al., 2011; **Figure 3A**, FCV). Notably, addition of No. 108 (45 μM) into the culture for 6 h yielded a BRET^2^ ratio equivalent to that of the No-virus sample (**Figure 3A**, No. 108), indicating inhibition of FCV protease activity in the cells. No cytopathic effects or morphological changes were observed by the 6 h treatment of No. 108 as monitored by light microscopy. The intensity of the RLuc signal representing the expression of the substrate protein was comparable between the No. 108-treated and -untreated cells under this treatment condition (12,524 and 11,664 counts per second, respectively). These results suggest that the No. 108 compound inhibited viral protease activity in the FCV-infected cells without significant damage to the cellular metabolism.

We next examined whether the compound No. 108 possesses anti-FCV protease activity *in vitro*. [^35^S]-labeled, His-tagged VP1 precursor protein of the FCV F4 strain and non-radiolabeled His-tagged NS6-7 protein containing protease and RdRp region of F4 strain (Oka et al., 2007) were separately expressed using *in vitro* transcription-translation system (Oka et al., 2009). Translational products were purified with MagZ Protein Purification System (Promega), mixed, incubated at 30°C for 20 h, and separated by SDS-polyacrylamide gel electrophoresis (Oka et al., 2009). The compound No. 108 showed significant activity against F4 protease in a dose dependent manner as indicated by a gradual decrease in the VP1 precursor cleavage products, i.e., mature VP1 and the leader capsid (**Figure 3B**, left panel). Similar changes were seen in the samples with the No. 108 derivative (compound No. 731), although higher concentrations of the No. 731 seemed to induce aggregation of the translational products as indicated by the increased signals in the origin of the gel (**Figure 3B**, middle panel). Such changes in processing profiles were not detected with the solvent of these chemical compounds (**Figure 3B**, right panel).

**FIGURE 4:**
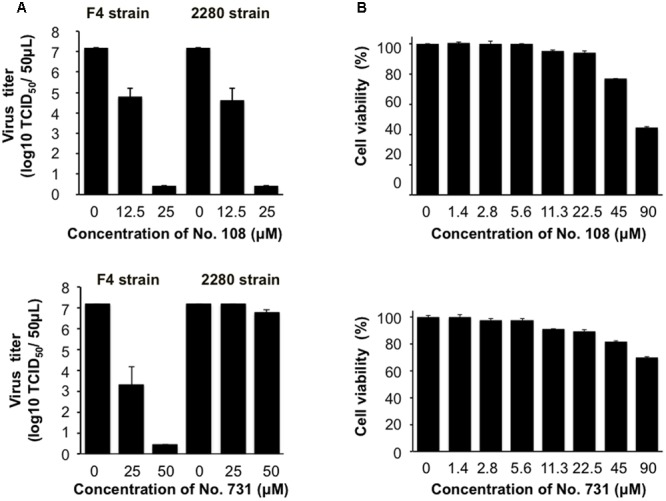
Evaluation of anti-FCV activity of the candidate molecules. **(A)** Dose-dependent inhibitory effect of compound No. 108 and No. 731 on the FCV growth in CRFK cells. CRFK cells were infected with the FCV F4 strain or FCV 2280 strain at a MOI of 0.0001. The cell culture supernatants were collected at 40 h post-infection in the presence of compound No. 108 (12.5 or 25 μM) or DMSO only, and the virus titer was determined as TCID_50_/50 μL. Upper panel: compound No. 108 that has structural portions mimicking the P3 and P4 sites of authentic substrate. Lower panel: compound No. 731 that is a No. 108 derivative that has structural portion mimicking the P3 site. **(B)** Evaluation of the cytotoxicity of compound No. 108 and No. 731. The cytotoxic effects of the No. 108 on the CRFK cells were measured by a CellTiter Glo Luminescent Cell Viability Assay Kit (Promega) at 48 h after compound treatment.

We further examined whether the compound No. 108 possesses anti-FCV activity in the cells by using two infectious FCV strains, FCV F4 (Oka et al., 2007) and 2280 (Oka et al., 2013). FCV-susceptible CRFK cells were incubated for 2 h with these viruses, washed, and incubated in the presence or absence of No. 108. Notably, an approximately 100-fold reduction in viral infectivity was detected for both viruses by the treatment with 12.5 μM of No. 108, and complete elimination of the infectivity was observed with 25 μM of No. 108 (**Figure 4A**, upper panel). In contrast, the compound No. 731 showed antiviral activity only against F4 strain (**Figure 4A**, lower panel). Compounds No. 108 and No. 731 induced significant cytotoxicity at a concentration of 90 μM, whereas the reduction in the number of viable cells was less than 5% and 10% at 22.5 μM of No. 108 and No. 731, respectively (**Figure 4B**). In sum, these results suggest that the No. 108 compound could specifically inhibit FCV replication at around a concentration of 12.5 μM.

### Binding Modes of No. 108 to FCV Proteases

**FIGURE 5:**
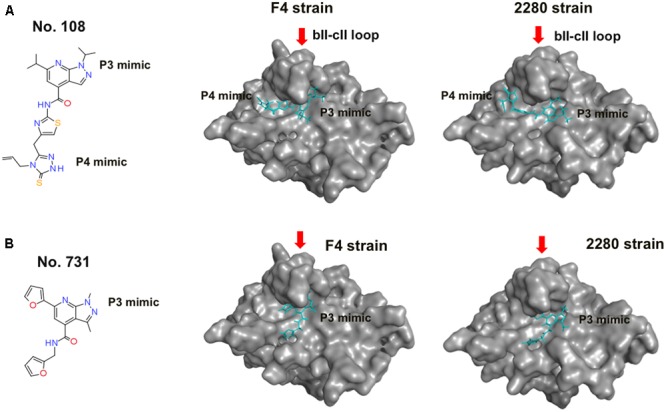
Binding modes of No. 108 and No. 731 to FCV proteases. **(A)** No. 108. **(B)** No. 731. Left panels: molecular formulas of the compound. Middle and Right panels: structural models of the FCV proteases docked to each compound. Each compound was docked with the protease domain of the FCV F4 strain (Oka et al., 2007) (GenBank accession number: D31836) or the 2280 strain (Oka et al., 2013) (Genbank accession no. KC835209) using the automated ligand docking program ASEDock 2005 (Goto et al., 2008) operated in MOE as described previously (Yokoyama et al., 2012b). The structures with the best docking score expressed by the arbitrary docking energy (U_dock_) in ASEDock2005 are shown. Red arrows indicate the bII-cII loop (Fernandes et al., 2015) near the substrate binding site. No. 108 is shown using a cyan stick model.

Compound No. 108 had structural portions that reasonably resembled the P3 and P4 sites, and are arranged in a 3-D position similar to that of the authentic substrate (**Figure 5A**, left panel). However, its binding mode was an open question, because the pharmacophore-based screening searches for the structural mimics of the substrate in the complex, yet does not address their binding modes. To obtain structural insights into the FCV protease-compound interactions, we constructed 3-D models of intact protease domains that were docked to the No. 108 compound (see Materials and Methods for details). We constructed two complex models for the FCV F4 and 2280 strains. The identity of the protease regions of the two FCV strains was about 97.4%: 150 out of 154 amino acid residues were identical. Despite the variation, the docking positions of the No. 108 in the proteases were very similar (**Figure 5A**, middle and right panels): in both cases, the No. 108 was placed in the same orientation in a protease cleft near a loop, termed the bII-cII loop (Fernandes et al., 2015). The docking positions of the No. 108 were consistent with the pharmacophore map constructed on the basis of the docking model between FCV F4 protease and its authentic substrate octapeptide (**Figure 2B**). Other docking positions yielded docking results with poor docking scores as assessed by the arbitrary docking energy (U_dock_) in ASEDock2005. Compound No. 731 is a derivative of the compound No. 108 and had structural portion that resembled the P3 site of authentic substrate (**Figure 5B**, left panel). The docking positions of the No. 731 in the proteases were different from those of the No. 108, although the P3 mimic portion was placed in the similar site near the bII-cII loop (**Figure 5B**, middle and right panels). In sum, these results suggest that the No. 108 and No. 731 have physicochemical features that allow them to bind to FCV proteases, although binding mode is different.

### MD Simulation of the Protease-Substrate Complex Model

**FIGURE 6:**
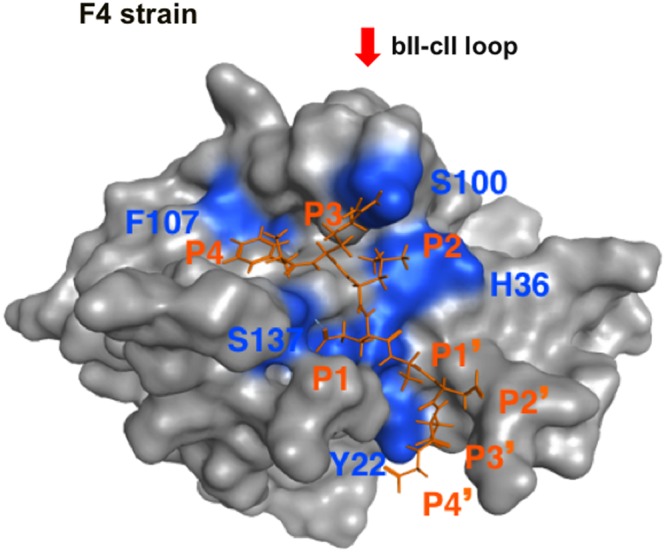
MD simulation of the protease-substrate complex model. The molecular model of the FCV F4 strain protease-substrate complex was subjected to MD simulation using modules in the AMBER program package (Case et al., 2014) as described for MD simulations of the HIV-1/SIV envelope gp120 subunit (Naganawa et al., 2008; Yokoyama et al., 2012a, 2016; Kuwata et al., 2013; Hikichi et al., 2016). After heating calculations for 20 ps until 310 K using the NVT ensemble for the constant volume, temperature, and numbers of particles in the system, simulation was executed using the NPT ensemble for the constant pressure, temperature, and numbers of particles in the system at 1 atm, at 310 K, and in 150 mM NaCl for 30 ns. The protease was restrained using a harmonic potential with a graded decreasing force constant from 10 to 1 kcal/mol/Å^2^. A structure at 30 ns of simulation is shown. A red arrow indicates the bII-cII loop (Fernandes et al., 2015) near the substrate binding site. The substrate octapeptide (FCV F4-6) is shown using an orange stick model.

The above results support the validity of our structural model for searching for a substrate mimic with antiviral activity. However, the initial model does not necessarily ensure fine resolution of molecular interactions, because it was constructed with the x-ray crystal structure and did not consider the dynamic aspects of the protein structure in solution. Therefore the model was further subjected to MD simulation to obtain a complex structure that may better approximate molecular structures and interactions in solution (Ode et al., 2012). The MD simulation showed that the substrate in the initial model settled into a thermodynamically more stable arrangement after 30 ns of the simulations, leading to re-positioning of the P4 residue to the site near the bII-cII loop (**Figure 6**). Importantly, the refined arrangement of the P3 and P4 residues in the FCV protease was strikingly similar to that of the P3 and P4 mimic portions of the No. 108 compound (**Figures 5, 6**).

### Three-Dimensional Map of Amino Acid Variations among FCV Proteases

**FIGURE 7:**
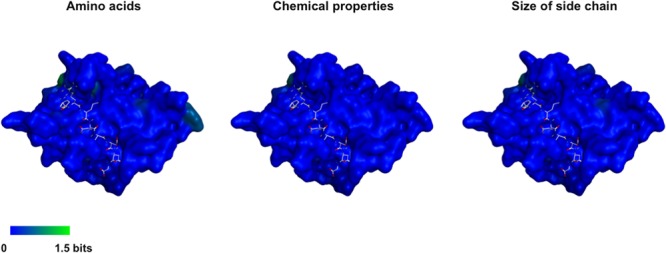
Three-dimensional map of the amino acid variations among FCV proteases. Shannon entropy scores based on amino acids **(Left)**, chemical properties of amino acids **(Middle)**, and size of side chains **(Right)**. Amino acid sequences of the protease domain (154 amino acid residues in length) (Sosnovtseva et al., 1999; Oka et al., 2007) of FCV strains for which complete genome sequences were available as of 2013 June were obtained from GenBank (*n* = 15, accession numbers: D31836, AF479590, U13992, M86379, AF109465, L40021, AY560118, AY560116, AY560117, AY560113, AY560114, AY560115, DQ424892, GU214989, and KC835209). Shannon entropy scores representing variations at individual amino acid positions of protein were calculated with a multiple sequence alignment as described previously for other viral proteins (Motomura et al., 2008; Naganawa et al., 2008; Oka et al., 2009). The entropy scores were expressed on the protease-octapeptide structure at 30 ns of MD simulation in **Figure 6**. The substrate octapeptide (FCV F4-6) is shown using a gray stick model. An entropy score of zero indicates absolute conservation, whereas that of 4.4 indicates complete randomness.

Using the MD-executed model, we examined amino acid variations among the interaction sites of FCV proteases. Shannon entropy representing variations at individual amino acid positions was calculated with the alignment of the protease domain sequences (*n* = 15) as described for other viral proteins (Motomura et al., 2008; Naganawa et al., 2008; Oka et al., 2009) and expressed on the 3-D model of the protease-substrate complex at 30 ns of MD simulations (**Figure 7**). The analysis showed that the FCV protease domain, including the binding cleft of the substrate and its mimic, is highly conserved among distinct strains. We noticed variations near the P3 and P4 interaction sites (entropy scores of 0.6–1.0) (**Figure 7**, left panel, greenish residues). However, when the entropy scores were calculated on the basis of chemical properties or the size of the amino acid residues (Oka et al., 2009; Yokoyama et al., 2012b), they were nearly zero across the substrate-binding cleft and its neighbors (**Figure 7**, middle and right panels). Together with the results in **Figure 6**, these data predicted that the authentic substrate and the No. 108 share the highly conserved binding site.

## Discussion

Unraveling the molecular interactions between a viral protease and its substrates is critical for understanding the molecular steps of viral replication and for developing antivirals. However, little is known about the interactions between the FCV protease and its substrates. In this report, we studied the binding mode of FCV protease and its substrate by molecular modeling and MD simulation. The structural information was then used to screen for a substrate-mimic compound, as was done in the screening for the anti-sapovirus compound (Yokoyama et al., 2012b; **Figure 2**). In this way, we identified an anti-FCV molecule, named No. 108. This compound has physicochemical properties and arrangement of the side chains at the P3 and P4 sites of the substrate in the protease-bound-state model (**Figures 2B, 5A**), has physicochemical features to bind to FCV proteases in a mode similar to the authentic substrate (**Figures 5, 6**), and has the ability to inhibit FCV protease activity *in vitro* and in the cells (**Figure 3**) and to suppress viral infectivity in the infected cells (**Figure 4**). Thus, the compound could be used to identify a substrate mimic with anti-FCV activity in FCV-infected cells. These results suggest the validity of our *in silico* model for elucidating protease-substrate interactions during viral replication and for developing antivirals.

The predicted binding mode of FCV protease and its substrate (**Figure 6**) was similar to that of norovirus protease and its substrate (Fernandes et al., 2015). In contrast, it was different from that of sapovirus protease and its substrate (Yokoyama et al., 2012b). These findings may reflect inherent structural features of the individual proteases, because the FCV and sapovirus proteases were both constructed using the crystal structure of Norwalk virus 3C-like protease. Thus, whereas calicivirus is classified into at least five groups on the basis of similarity of full-length genomes^1^, distinct grouping may be possible on the basis of the protease-substrate binding mode. Such a structure-based grouping would provide molecular insights into the classification of calicivirus proteases and for design antivirals. Further study involving *in silico* and experimental structural analyses will be needed to address this issue.

The MD-executed structural model may facilitate future structure-function study of the cleavage sites of the FCV precursor proteins. Viral precursor processing generally proceeds as an ordered, step-wise cascade, and disordered processing prevents particle assembly and maturation, leading to the elimination of viral infectivity, as typically seen in human immunodeficiency virus replication (Krausslich, 1991). Likewise, ordered processing seems to exist in replication of caliciviruses: the processing of the ORF1 precursor protein of norovirus (Belliot et al., 2003; May et al., 2013) and FCV (Sosnovtsev et al., 2002) is a step-wise process. However, it remains unknown whether and how the ordered processing plays roles in viral replication. It is possible that the sequence variation among the six cleavage sites of FCV precursors (**Figure 1**) causes variations in the substrate binding mode via distinct positioning of the scissile bonds of the substrates, water molecules, and catalytic residues of enzyme in the protease active center. These steric variations, if present, would cause variations in the hydrolysis efficiencies of peptide bonds and thereby lead to the ordered progression of viral replication. Our 3-D model in combination with the reverse genetics system of FCV (Sosnovtsev and Green, 1995; Oka et al., 2014; Sandoval-Jaime et al., 2015) will become important research tools for addressing each of these issues.

Our 3-D model will also help in elucidating the structural basis for FCV evolution. Despite the important roles played by the cleavage sites in calicivirus replication, these sites exhibit marked variations among strains (Oka et al., 2009). Considering the high level of conservation of the substrate interaction surface of FCV protease (**Figure 7**), the cleavage sites appear to play a key role in generating variants with distinct processing phenotypes, as seen in norovirus (Emmott et al., 2015). Generation of processing variants may be advantageous for generating variants with distinct replication fitness under the changing environments in cells and hosts. Our 3-D model in combination with the reverse genetics system of FCV will be useful for studying structure-phenotype relationship and the adaptive evolution of FCV.

Finally, the present findings have implications for the development of antivirals. The high level of conservation of the substrate interaction surface of protease suggests that a the strategy for intervening in protease-substrate interactions is feasible. Present and previous (Oka et al., 2009) studies show that much stronger constraints against amino acid changes are acting on viral protease than its substrates. Therefore, the substrate interaction sites of viral protease rather than the substrates themselves would be more reasonable targets for identifying conservative structural features to design better antivirals.

An important issue in future work would be the development of a new *in vitro* assay using a purified viral protease. Such a system would accelerate both the structure-function study of FCV protease and the development of antivirals in combination with the present *in silico* and BRET^2^ systems. Therefore, we preliminarily attempted to obtain a purified FCV protease domain that was composed of 154 amino acid residues in the carboxy-terminal region of the ORF1 (Sosnovtseva et al., 1999; Oka et al., 2007). However, the purifications somehow resulted in loss of the protease activity. Meanwhile, the expression and purification of the protease-polymerase fusion protein (**Figure 1**) resulted in a partial retention of protease activity (**Figure 3B**). Thus, the polymerase domain seems to play a role in increasing the stability of the active protease conformation. Being expressed as the protease-polymerase fusion form may also be significant for viral replication, because the fusion protein is stably expressed in FCV-infected cells (Sosnovtseva et al., 1999; Green et al., 2002). Taken together, our present investigation should provide a structural basis for future study of the FCV protease-polymerase fusion protein.

## Author Contributions

TiO and MY conceived and designed the study. MY performed molecular modeling and MD simulation. HK, TyO, and TN provided chemical compounds. TiO, HT, and YT performed the experiments. HS, TiO, and MY prepared the manuscript. All authors read and approved the final manuscript.

## Conflict of Interest Statement

The authors declare that the research was conducted in the absence of any commercial or financial relationships that could be construed as a potential conflict of interest.

## Footnotes

1 https://talk.ictvonline.org/ictv-reports/ictv_9th_report/positive-sense-rna-viruses-2011/w/posrna_viruses/253/caliciviridae
